# Effects of Single and Double Mutants in Human Glucose-6-Phosphate Dehydrogenase Variants Present in the Mexican Population: Biochemical and Structural Analysis

**DOI:** 10.3390/ijms21082732

**Published:** 2020-04-15

**Authors:** Víctor Martínez-Rosas, Merit Valeria Juárez-Cruz, Edson Jiovany Ramírez-Nava, Beatriz Hernández-Ochoa, Laura Morales-Luna, Abigail González-Valdez, Hugo Serrano-Posada, Noemí Cárdenas-Rodríguez, Paulina Ortiz-Ramírez, Sara Centeno-Leija, Roberto Arreguin-Espinosa, Miguel Cuevas-Cruz, Daniel Ortega-Cuellar, Verónica Pérez de la Cruz, Luz María Rocha-Ramírez, Edgar Sierra-Palacios, Rosa Angélica Castillo-Rodríguez, Isabel Baeza-Ramírez, Jaime Marcial-Quino, Saúl Gómez-Manzo

**Affiliations:** 1 Laboratorio de Bioquímica Genética, Instituto Nacional de Pediatría, Secretaría de Salud, Ciudad de México 04530, Mexico; ing_vicmr@hotmail.com (V.M.-R.); edsonjiovany@ciencias.unam.mx (E.J.R.-N.); lauraeloisamorales@ciencias.unam.mx (L.M.-L.); paulo.r1396@gmail.com (P.O.-R.); 2 Programa de Posgrado en Biomedicina y Biotecnología Molecular, Escuela Nacional de Ciencias Biológicas, Instituto Politécnico Nacional, Ciudad de México 11340, Mexico; beatrizhb_16@comunidad.unam.mx; 3 Departamento de Ingeniería Bioquímica, Posgrado en Ciencias de los Alimentos, Escuela Nacional de Ciencias Biológicas, Instituto Politécnico Nacional, Ciudad de México 11340, Mexico; juarezmerit@gmail.com; 4 Posgrado en Ciencias Biológicas, Universidad Nacional Autónoma de México, Ciudad de México 04510, Mexico; 5 Laboratorio de Inmunoquímica. Hospital Infantil de México Federico Gómez, Secretaría de Salud, Ciudad de México 06720, Mexico; 6 Departamento de Biología Molecular y Biotecnología, Instituto de Investigaciones Biomédicas, Universidad Nacional Autónoma de México, Ciudad de México 04510, Mexico; abigaila@biomedicas.unam.mx; 7 Consejo Nacional de Ciencia y Tecnología (CONACYT), Laboratorio de Agrobiotecnología, Tecnoparque CLQ, Universidad de Colima, Carretera los Limones-Loma de Juárez, Colima 28629, Mexico; hjserranopo@conacyt.mx (H.S.-P.); scenteno0@ucol.mx (S.C.-L.); 8 Laboratorio de Neurociencias, Instituto Nacional de Pediatría, Secretaría de Salud, Ciudad de México 04530, Mexico; noemicr2001@yahoo.com.mx; 9 Departamento de Química de Biomacromoléculas, Instituto de Química, Universidad Nacional Autónoma de México, Ciudad de México 04510, Mexico; arrespin@unam.mx (R.A.-E.); miguel.ccqi@yahoo.com.mx (M.C.-C.); 10 Laboratorio de Nutrición Experimental, Instituto Nacional de Pediatría, Secretaría de Salud, Ciudad de México 04530, Mexico; dortegadan@gmail.com; 11 Departamento de Neuroquímica, Instituto Nacional de Neurología y Neurocirugía Manuel Velasco Suárez, S.S.A., Ciudad de México 14269, Mexico; veped@yahoo.com.mx; 12 Departamento de Infectología, Hospital Infantil de México Federico Gómez, Delegación Cuauhtémoc, Ciudad de México 06720, Mexico; luzmrr7@yahoo.com.mx; 13 Colegio de Ciencias y Humanidades, Plantel Casa Libertad, Universidad Autónoma de la Ciudad de México, Ciudad de México 09620, Mexico; edgar.sierra@uacm.edu.mx; 14 Consejo Nacional de Ciencia y Tecnología (CONACYT), Instituto Nacional de Pediatría, Secretaría de Salud, Ciudad de México 04530, Mexico; racastilloro@conacyt.mx; 15 Laboratorio de Biomembranas. Escuela Nacional de Ciencias Biológicas, Instituto Politécnico Nacional, Ciudad de México 11340, Mexico; isabelbaeza@yahoo.com

**Keywords:** human G6PD mutants, G6PD deficiency, protein stability, structural characterization, catalytic activity

## Abstract

Glucose-6-phosphate dehydrogenase (G6PD) deficiency is the most frequent human enzymopathy, affecting over 400 million people globally. Worldwide, 217 mutations have been reported at the genetic level, and only 19 have been found in Mexico. The objective of this work was to contribute to the knowledge of the function and structure of three single natural variants (G6PD A+, G6PD San Luis Potosi, and G6PD Guadalajara) and a double mutant (G6PD Mount Sinai), each localized in a different region of the three-dimensional (3D) structure. In the functional characterization of the mutants, we observed a decrease in specific activity, protein expression and purification, catalytic efficiency, and substrate affinity in comparison with wild-type (WT) G6PD. Moreover, the analysis of the effect of all mutations on the structural stability showed that its presence increases denaturation and lability with temperature and it is more sensible to trypsin digestion protease and guanidine hydrochloride compared with WT G6PD. This could be explained by accelerated degradation of the variant enzymes due to reduced stability of the protein, as is shown in patients with G6PD deficiency.

## 1. Introduction

Glucose-6-phosphate dehydrogenase (G6PD) (EC 1.1.1.49) is a cytosolic enzyme that catalyzes the conversion of β-D-glucose-6-phosphate to 6-phosphoglucono-δ-lactone with the concomitant production of a reduced form of nicotinamide adenine dinucleotide phosphate (NADPH). This reaction is the first step in the pentose phosphate pathway, which provides the cells with reduced equivalents that are used in the biosynthesis process and counteract oxidative stress [1]. NADPH is necessary to maintain the glutathione on a reduced form with the enzyme glutathione reductase which gives protection against high physiologic levels of oxidant agents (free radicals and peroxides) [2]. In the erythrocytes, this defense is given by the glutathione antioxidant system, in which the enzyme G6PD allows this environment to be kept reduced and favors hemoglobin and other proteins [3].

G6PD deficiency, a hereditary genetic defect, is the most common enzymopathy in humans, affecting more than 400 million people worldwide, indicating a global prevalence of 4.9% [4]. The prevalence of G6PD deficiency correlates with the geographical distribution of endemic areas malaria, and has been postulate that G6PD deficiency is an adaptative response as protection against severe forms of *P. falciparum* malaria and that inheriting the G6PD deficiency gene reduces the severity of malaria infection [5,6,7,8]. The clinical spectrum of this disease includes patients with no symptoms to those with neonatal hyperbilirubinemia resulting in kernicterus (which can be fatal), episodes of acute hemolysis due to the ingestion of exogenous agents (drugs, food, or infectious agents), or chronic nonspherocytic hemolytic anemia [3,9].

G6PD deficiency is usually diagnosed and classified based on its residual enzyme activity and the patient’s hematologic parameters, ranging from the most severe manifestations with less than 5% of residual activity (Class I) to the mildest form (Class V) [10]. Worldwide, 217 mutations have been reported, and most of the reported variants are single-point mutations (83.9%) causing a single amino acid substitution. Double and triple mutants have also been reported (8.7%), but with lower frequencies, which indicates that this disease is heterogeneous [11,12]. Regrettably, only about 10% of G6PD variants have been studied at the level of structural and functional characterization.

In Mexico, the presence of 19 different mutants has been reported, which have been found in several parts of the country, including single nucleotide substitutions (missense variants) G6PD A+ (Asn126Asp), G6PD San Luis Potosi (Asn126Tyr), and Guadalajara (Arg387Cys), which have given rise to the double mutant G6PD Mount Sinai (Asn126Asp + Arg387Cys) [13,14]. It is important to note that the Class II G6PD San Luis Potosi and two Class I G6PDs, Guadalajara and Mount Sinai variants, have not been functionally and biochemically characterized.

**Figure 1:**
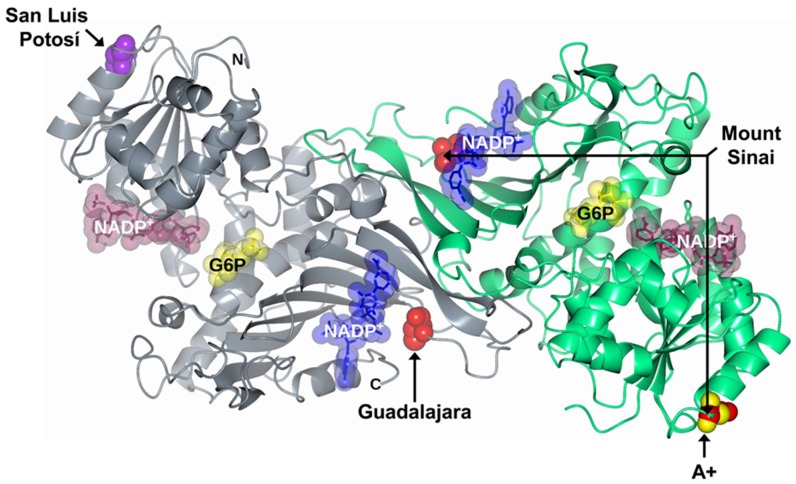
Structure of glucose-6-phosphate dehydrogenase (G6PD) dimer (Protein Data Bank entries 2BHL and 2BH9) indicating location of Class I mutations Guadalajara (R387C) and Mount Sinai (N126D + R387C) (red spheres), Class II mutation San Luis Potosi (N126Y) (purple spheres), and Class III mutation A+ (N126D) (yellow spheres). Structural nicotinamide adenine dinucleotide phosphate (NADP+), catalytic NADP+, and glucose-6-phosphate (G6P) are drawn in blue, dark purple, and yellow molecular surface representations, respectively. Monomers are shown in slate gray and spring green. Same color code is used in all other figures.

Previously we studied the Class III G6PD A+ variant (single nucleotide substitutions) and also characterized it as a comparative variant with respect to G6PD San Luis Potosi, in which the single nucleotide substitutions are located in the same 126 amino acid codon. As previously noticed, the G6PD A+ variant was related in an asymptomatic G6PD deficiency form [6]. This mutation results in a change of adenine by guanine (A→G) in nucleotide (nt) 376 of the G6PD gene and is located in exon 5, with a change on the asparagine by aspartic acid (Asn→Asp) amino acid residue in the 126 position of the three-dimensional (3D) structure (Figure 1). It is interesting to note that the mutation is far from both the active site and the interface region of the protein; nevertheless, the asparagine 126 in the wild-type form of the enzyme is part of the α-helix structure exposed in an aqueous medium [15,16]

Class II G6PD San Luis Potosi was reported for the first time in a study which included 5000 Mexican individuals, where the G6PD deficiency was determined in the Mexican population. This study was carried out across the country and the variant was detected in an anonymous blood sample from San Luis Potosi State. This mutant showed a single nucleotide substitution of adenine for thymine (A→T) at nt 376 (exon 5), which results in the substitution of asparagine by tyrosine 126 (Asn→Tyr) amino acid residue [17] (Figure 1). This variant showed that the mutation is localized in the same position (nt 376) as the G6PD A+ variant [15]. The single Class I G6PD Guadalajara variant shows the substitution of cytosine by thymine (C→T) at nt 1159 (exon 10), which gives a change in the arginine residue by cysteine 387 (Arg→Cys) located in the binding site of structural NADP+ of the 3D structure, which is implicated in enzyme stability and dimer formation. This mutant was detected in a 3-year-old boy born in Guadalajara (Mexico) who presented with neonatal jaundice, hemoglobinuria, and chronic nonspherocytic hemolytic anemia, requiring blood transfusions [10,18].

Finally, the double mutant G6PD Mount Sinai (A376G/C1159T) (Asn126Asp + Arg387Cys) involves the mutations G6PD A+ and G6PD Guadalajara (Figure 1). This double mutant is classified as a Class I variant because patients show neonatal jaundice, hemoglobinuria, and chronic nonspherocytic hemolytic anemia, requiring blood transfusions [10,16]. Furthermore, it has been observed that patients with this double mutant show residual glucose-6-phosphate dehydrogenase activity of around 10% [16].

In this study, from the solved three-dimensional structure of the human G6PD protein, we defined changes in the protein structure to explain the clinical manifestations of these mutations and performed biochemical studies of the single natural variants (G6PD A+, G6PD San Luis Potosi, and G6PD Guadalajara) and the double mutant Class I G6PD Mount Sinai (G6PD A+ plus G6PD Guadalajara) to fully understand the molecular mechanisms underlying the observed clinical manifestations observed in individuals with G6PD mutations. All the natural mutants were constructed, overexpressed, and purified, and thermostability assays and detailed steady-state kinetics were determined even though they had little activity. Finally, we found again that mutations in the G6PD gene classified as a Class II variant exhibit loss of catalysis and were similarly unstable as the previous values obtained for Class I mutants.

## 2. Results and Discussion

### 2.1. In Silico Mutagenesis and Modeling

**Figure 2:**
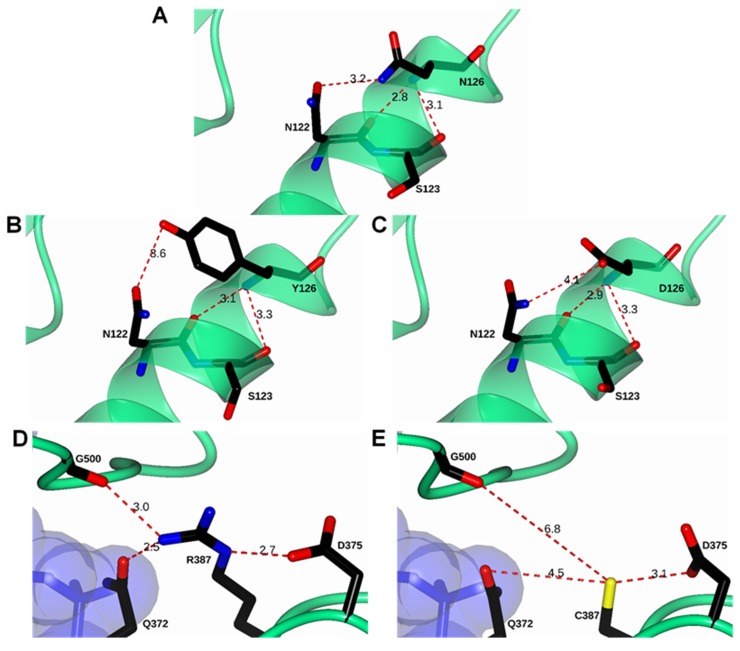
Structural comparison between human glucose-6-phosphate dehydrogenase (G6PD) enzyme (PDB entries 2BHL and 2BH9) and minimized models of Class I Guadalajara and Mount Sinai, Class II San Luis Potosi, and Class III A+. (**A**) Wild-type (WT) G6PD enzyme; (**B**) in silico N126Y mutation; (**C**) in silico N126D mutation; (**D**) WT G6PD enzyme; and (**E**) in silico R387C mutation. Note that double mutant R387C/N126D Mount Sinai is shown in (**C**,**E**). Residues are shown as black cylinders. Distances are in Å.

To understand the effect of the mutations on the structure of glucose-6-phosphate dehydrogenase (G6PD) dimer, we performed in silico mutagenesis and modeling. When the asparagine residue was changed to tyrosine in position 126 to give the single natural variant G6PD San Luis Potosi, we observed that the mutation occurred on β-helices exposed to an aqueous medium. We noticed three interactions: The first of 3.2 Å between the nitrogen of the amino group of N126 with the lateral chain oxygen of the residue N122; a second interaction (2.8 Å) between the amino group of the peptide bond of N126 and the oxygen on the functional carbonyl group N122; and a third interaction (3.1 Å) between the carbonyl group S123 and the nitrogen on the peptide bond of N126 on the wild-type G6PD human enzyme (Figure 2A). When the asparagine residue (N126) was replaced by tyrosine (Y126) (Figure 2B), we noticed a greater distance between amino acids N122 and S123 weakening these interactions, making the area more flexible and lose part of the alpha-helix spin, which could cause the enzyme to destabilize and lose more than 90% of their catalytic activity, although that this mutation is far from the active site or the structural NADP^+^ binding site.

On the other hand, when we performed the analysis in silico and the arginine residue was changed to cysteine in position 387 of the polypeptide chain (G6PD Guadalajara), we noticed that this change resulted in an increase of the distance of the interactions between the nitrogen of the lateral chain of R387 and the oxygen of carbonyl group Q372 (2.5 Å) and the interaction between the nitrogen of the lateral chain of R387 with the oxygen of lateral chain G500 (3.0 Å) of the wild-type human enzyme (Figure 2D). in the same way, when the arginine residue (R387) was changed by cysteine (C387) (Figure 2E), the mutation occurred near the interface of the dimer, localized on the union site to the NADP^+^ structure, therefore, with the change by cysteine, the increased distance of these interactions could be the cause of the low stability and conformation structure of the enzyme, reflected in the loss of catalytic activity (more than 90%), causing the disease phenotype.

Finally, we noticed that the individual mutants G6PD A+ and G6PD Guadalajara gave rise to the double mutant Mount Sinai. As mentioned previously, for each mutant, the increased distance between interactions by N126 (Figure 2C) and R387C (Figure 2E) resulted in a loss of more than 90% of the catalytic activity and the structural activity of the protein, reflected in the most severe manifestation of the disease.

### 2.2. Construction Expression and Purification of Recombinant G6PD Variants 

**Figure 3:**
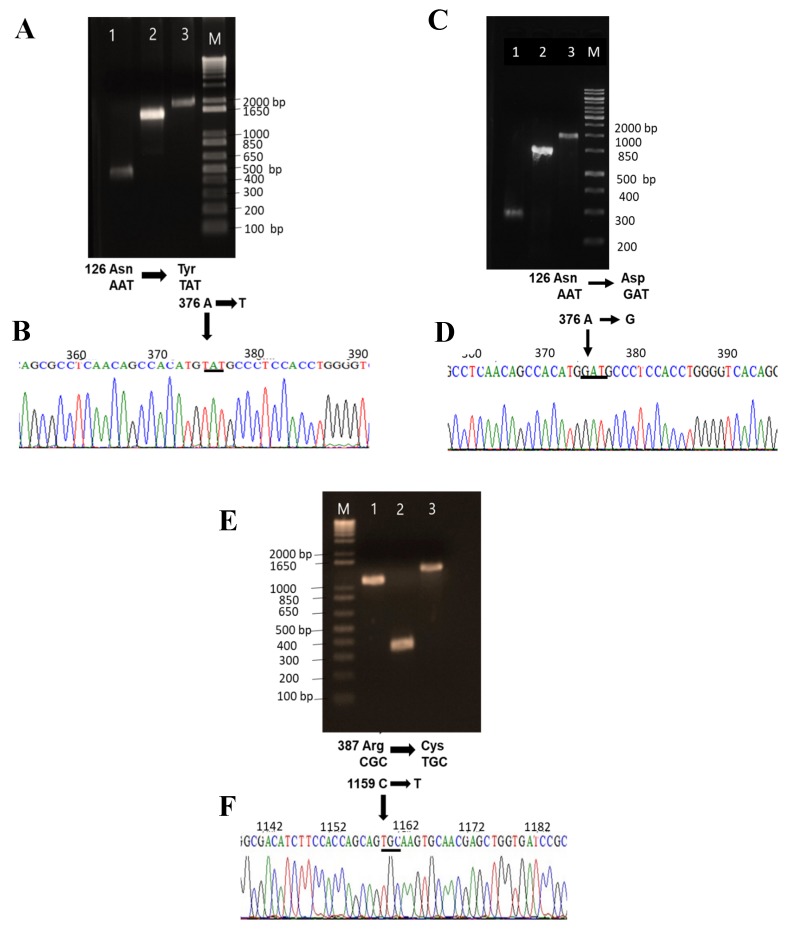
Site-directed mutagenesis for obtaining G6PD variants. (**A**) Amplicon resulting from polymerase chain reaction (PCR) in agarose gel electrophoresis (1%) for G6PD variant San Luis Potosi. Lanes 1 and 2. G6PD San Luis Potosi fragments. (**B**) Electropherogram of G6PD San Luis Potosi (A376, Asn→Tyr). (**C**) Variant G6PD A+. Lanes 1 and 2 G6PD A+ fragments. (**D**) Electropherogram of G6PD A+ (A376G, Asn→Asp). (**E**) Variant G6PD Guadalajara. Lanes 1 and 2 G6PD Guadalajara fragments. (**F**) Electropherogram of G6PD Guadalajara (C1159T, Arg→Cys). Line marked M in agarose gel electrophoresis corresponds to the Marker O’GeneRuler 1kb DNA ladder (Thermo Scientific) and line marked 3 corresponds to amplification of full-length G6PD.

The three single clinical mutants, G6PD A+ (N126D), G6PD San Luis Potosi (N126Y), and G6PD Guadalajara (R387C), and one double mutant, G6PD Mount Sinai (N126D/R387C), were created by site-directed mutagenesis (Figure 3). The incorporation of the desired mutation in the G6PD gene was corroborated by bidirectional DNA sequence analysis in the constructed plasmids (pJETR387C, pJETN126Y, pJETN126D, and pJETN126D/R387C). As can be observed in the electropherograms of the single mutant G6PD San Luis Potosi, a single nucleotide substitution of adenine for thymine (A→T) at nt 376 ws observed (Figure 3B); while for G6PD A+ the adenine was replaced by guanine (A→G) in nucleotide (nt) 376 (Figure 3D). Finally, in the G6PD Guadalajara variant a substitution of cytosine by thymine (C→T) at nt 1159 was introduced (Figure 3F). The recombinant G6PD mutants with the desired mutations (Figure 3) were sub-cloned into the pET3a plasmid and transformed into competent E.coli BL21 (DE3) zwf::kanr cells to produce recombinant G6PD variants.

**Figure 4:**
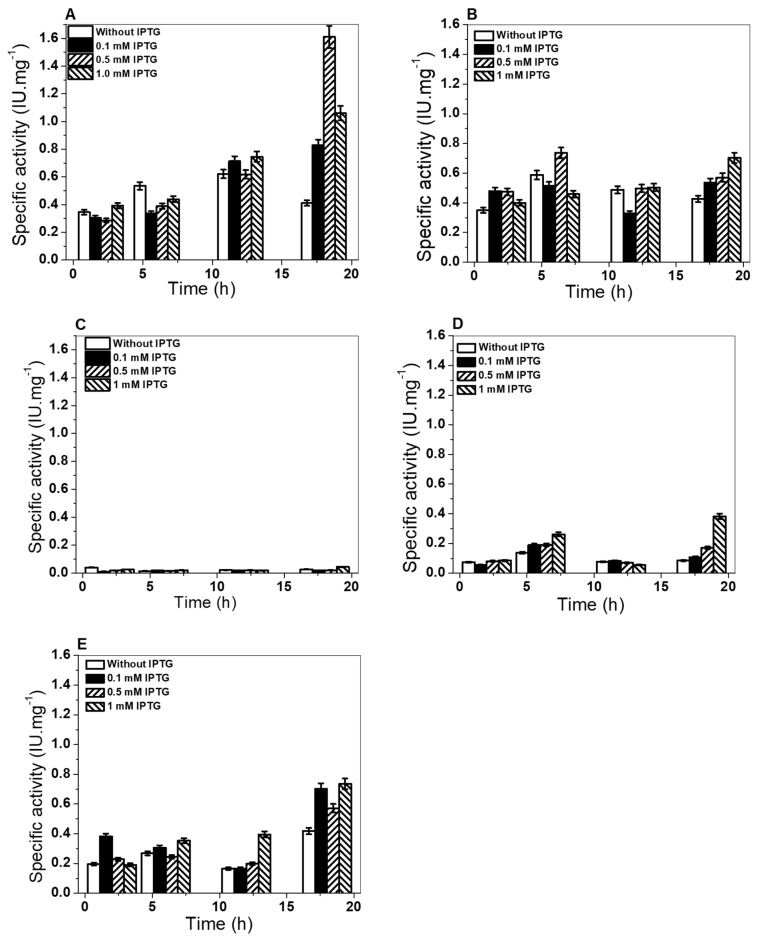
Overexpression of G6PD variants in a heterologous system. (**A**) G6PD WT human recombinant; (**B**) G6PD single mutant A+; (**C**) G6PD San Luis Potosi; (**D**) G6PD Guadalajara; (**E**) double G6PD mutant Monte Sinai. Crude extract was measured at 340 nm to calculate specific activity based on the concentration of protein of each culture. Assays were performed in triplicate.

To characterize the single mutants G6PD A+ (Asn126Asp), G6PD San Luis Potosi (Asn126Tyr), and Guadalajara (Arg387Cys) and the double mutant G6PD Mount Sinai (A Asn126Asp + Arg387Cys), we first determined the optimal conditions of soluble proteins using specific activity as an indicator. Also, we compared it against the WT G6PD. In these assays, the cells were induced with different conditions of isopropyl-β-D-tiogalactopiranoside (IPTG) at different incubation times, as has been reported by Gomez-Manzo et al. [19]. As shown in Figure 4B, for the G6PD A+ variant, specific activity of 0.75 UI*mg^−1^ was obtained with 0.5 mM of IPTG; meanwhile, for the G6PD San Luis Potosi variant (Figure 4C), specific activity of 0.064 UI*mg^−1^ was obtained from crude extract with 1 mM of IPTG, reaching maximum specific activity at 6 and 18 h of incubation of both enzymes.

On the other hand, specific activity of 0.4 UI*mg^−1^ was obtained with 1 mM of IPTG at 18 h for the G6PD Guadalajara variant (Figure 4D); for the G6PD Monte Sinai variant, specific activity of 0.75 UI* mg^−1^ was obtained with 1 mM of IPTG at 18 h (Figure 4E). As expected, the expression of the four mutants was less than the WT G6PD enzyme (1.6 UI*mg^−1^) (Figure 4A). An approximate 2.1-, 25-, 4-, and 2.1-fold decrease in specific activity of the crude extract was obtained from G6PD A+, G6PD San Luis Potosi, G6PD Guadalajara, and G6PD Mount Sinai, respectively, with respect to the WT G6PD enzyme. It is important to note that the level of expression of the three single and one double G6PD mutants is less, although the mutations are localized in different parts of the three-dimensional structure of WT G6PD protein. However, this low expression of soluble protein is similar to other variants, such as G6PD Mexico, G6PD Seattle, and G6PD Veracruz. Using the best overexpression conditions, we purified the G6PD proteins (WT and variants) by affinity and anionic interchange columns. The analysis of the purity of the enzymes was performed in 12% acrylamide gel (SDS–PAGE), where a single band (96% purity) was observed for the WT and the variants, which allowed us to carry out the experimental and structural trials. The purification results are summarized in Table 1; the chromatographic steps resulted in protein concentrations between 0.4 and 2.5 mg (per 2 L of *E*. *coli* culture), however the purification efficiency of the four variants was lower with respect to WT G6PD enzyme. Finally, it is interesting to note that we need 5-fold total protein (1 µg total protein) to be able to measure specific activity of single mutants G6PD San Luis Potosi, G6PD Guadalajara, and double mutant G6PD Mount Sinai with respect to WT G6PD (200 ng of total protein). The yield (%) and total protein obtained in the purification of each G6PD mutant again indicate that even though the mutations were located in different regions of the three-dimensional structure (Figure 1), they had a negative effect on G6PD expression (Figure 4) and purification, which could be related to the lower stability of the mutant proteins.

### 2.3. Determination of Steady-State Kinetic Parameters

**Figure 5:**
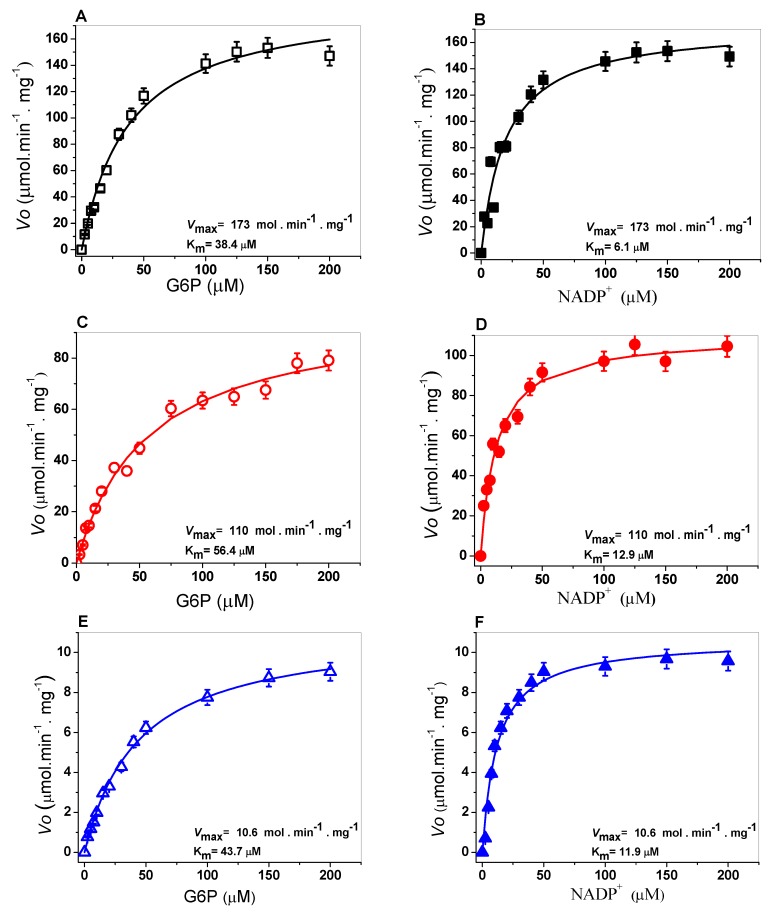

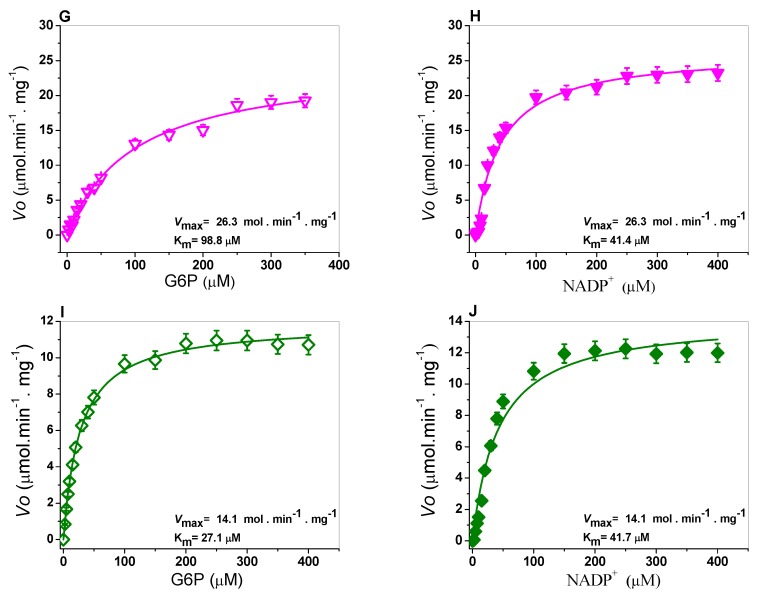
Kinetic characterization of (**A**,**B**) human G6PD WT and three single mutants: (**C**,**D**) G6PD A+ (Asn126Asp), (**E**,**F**) G6PD San Luis Potosi (Asn126Tyr), (**G**,**H**) G6PD Guadalajara (Arg387Cys); and (**I**,**J**) double mutant G6PD Monte Sinai. Initial velocities (*V*o) of each concentration of G6PD physiological substrate (G6P and NADP^+^) were fitted to the Michaelis–Menten equation by nonlinear regression calculation to obtain V_max_ and K_m_ values. All assays were performed in triplicate.

The kinetic parameters were determined to evaluate catalytic efficiency and analyze the effects of the mutations in the G6PD A+ (N126D), San Luis Potosi (N126Y), Guadalajara (R387C), and G6PD Mount Sinai (N126D/R387C). As can be observed in Figure 5, the WT G6PD and the four variants showed hyperbolic behavior for both substrates (G6P or NADP^+^), therefore, the initial velocity values obtained at the substrate concentrations (NADP+ and G6P) were adjusted to the Michaelis–Menten equation by nonlinear regression.

Steady-state kinetic parameters were obtained from the plots and are summarized in Table 2. The K_m_ value for the NADP^+^ and G6P substrates of the WT G6PD enzyme was 6 µM and 38 µM, respectively (Figure 5A,B). The *K_m_* value of the physiological substrates on variant G6PD A^+^ (Class III) was K_m G6P_ = 56.4 µM (Figure 5C), and a 1.4-fold increase was shown concerning the WT G6PD enzyme, and for K_m NADP_^+^ = 12.9 µM (Figure 5D) there was a 2.1-fold increase in substrate affinity. This resulted in a 50% decrease in the catalytic constant (*k*_cat_ = 114 s^−1^) with respect to WT G6PD.

On the other hand, for G6PD San Luis Potosi, K_m NADP+_ = 11.9 µM (Figure 5F) was approximately 2-fold higher compared to the WT G6PD enzyme; while the G6P substrate (K_m G6P_ = 43.7 µM) (Figure 5E) was 1.1-fold higher than the WT G6PD enzyme. The catalytic constant (*k*_cat_) decreased by more than 95% (*k*_cat_ = 10.4 s^−1^) with respect to the WT G6PD enzyme (*k*_cat_ = 230 s^−1^). It is interesting to note that a 10-fold loss in the catalytic constant (*k*_cat_) was observed in the mutant G6PD San Luis Potosi (N126Y) with respect to G6PD A+ (N126D), where both mutations are located in the same codon, and the replacement of asparagine amino acid residue, giving that one mutant was reportedly Class III and the other Class II.

Furthermore, as seen in Figure 5, the corresponding affinity values for the G6PD Guadalajara enzyme (Class I) for both substrates (NADP^+^ and G6P) were K_m NADP_ = 41.4 µM (Figure 5H) and K_m G6P_ = 98.8 µM (Figure 5G). These values show an approximately 7-fold loss of affinity of the enzyme for its substrates for the NADP^+^ substrate and 2.6-fold for the G6P substrate, resulting in a loss of catalysis of more than 90% (*k*_cat_ = 25.9 s^−1^) with respect to the WT G6PD enzyme (*k*_cat_ = 230 s^−1^).

Finally, saturation curves for the double mutant G6PD Mount Sinai (Class I) were performed and the kinetic parameters generated by the double mutation in the protein were determined. A decrease in the catalytic constant of 94% (*k*_cat_ = 13.9 s^−1^) was observed. Furthermore, the affinity values determined for the substrates NADP^+^ and G6P were lower K_m NADP_^+^ = 41.7 µM (Figure 5J) and K_m G6P_ = 27.1 µM (Figure 5I) concerning the WT G6PD enzyme.

Finally, it is important to mention that although G6PD San Luis Potosi has been classified as a Class II mutant according to patients’ hematological parameters, the recombinant G6PD variant showed a loss of catalysis similar to the previous values obtained for Class I mutants. The steady-state kinetic parameters obtained for the single mutant Class I G6PD Guadalajara and double mutant Class I G6PD Mount Sinai are similar to those reported for variants such as G6PDs Volendam, Andalus, Nashville, Durham, and Zacatecas, which lost nearly 90% of the catalytic constant (*k*_cat_) with respect to WT GPD enzyme, which could explain the lack of reduced form of NADPH and counteract the oxidative stress on red blood cells, hence resulting in the most severe manifestation of the disease, chronic nonspherocytic hemolytic anemia [20,21,22]. The reclassification of the G6PD San Luis Potosi variant could allow a targeted treatment to the clinical hemolytic episodes of the patients with this G6PD mutation (in particular on malaria treatment), provide more information of the hemolytic risk and explain the severe clinical phenotypes by biochemical alterations on G6PD protein of these individuals.

### 2.4. Circular Dichroism (DC) Analysis

**Figure 6:**
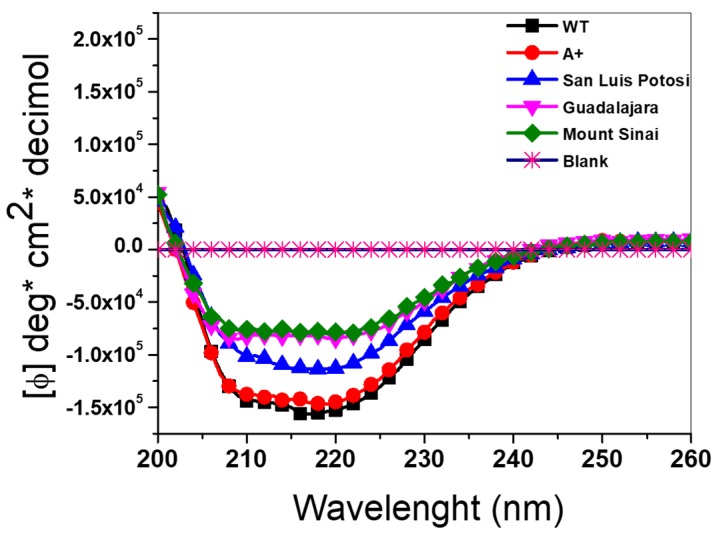
Circular dichroism (DC) spectra of four recombinant variants of human G6PD. Structural characterization was performed in the far UV region (200–260 nm). The experiment was conducted in triplicate.

Circular dichroism (DC) assay was performed to determine if the loss in catalytic activity of the four recombinant G6PD variants was due to alterations in the secondary structure provoked by the change in amino acid residue in each variant. Figure 6 shows the spectra of the three single mutants G6PD A+ (N126D), San Luis Potosi (N126Y), and Guadalajara (R387C) and the double mutant G6PD Mount Sinai (N126D/R387C) plus the WT enzyme. We observed that all G6PD mutants had an absorption pattern at 208 and 222 nm, reflecting a protein with α-β conformation similar to the WT G6PD. However, all G6PD mutants (except for the A+ variant) showed minimum absorption signals, which indicates that an effect in the secondary structure caused by these mutations was observed as a change in absorption of the variants with respect to the absorption of the WT G6PD enzyme on the molar ellipticity (ϕ) in spectra of 222 nm and 208 nm for the α−helices and β−sheets, respectively, which may explain the decrease in catalytic efficiency.

It was observed that the effect of the mutations in the secondary structure of G6PD were proportional to the loss of catalytic activity, where the single G6PD A+ variant showed catalytic activity around 85% with respect to WT G6PD enzyme, and no alterations in the secondary structure were observed. However, G6PD San Luis Potosi, G6PD Guadalajara, and the double mutant G6PD Mount Sinai lost more secondary structure, with G6PD Mount Sinai presenting the greatest loss of structure. It is interesting to note that the presence of the mutation of the variant Guadalajara (R387C) in the double mutant G6PD Mount Sinai (N126D/R387C) is the main factor responsible for the alteration in the secondary structure of double mutant G6PD Mount Sinai, because the spectrum of the single A+ variant (present in G6PD Mount Sinai) was very close to that recorded by the WT G6PD enzyme. In addition, the catalytic activity of G6PD Guadalajara was less with respect to the G6PD A+ variant. Therefore, it is possible to propose an additive effect of the G6PD Guadalajara variant on the G6PD A+ variant, giving the double mutant G6PD Mount Sinai greater alteration of the secondary structure and greater loss in catalytic activity with respect to the WT G6PD enzyme.

### 2.5. Structural Analysis by Intrinsic and 8-Anilinonaphthalene-1-Sulfonate (ANS) Binding Assays

**Figure 7:**
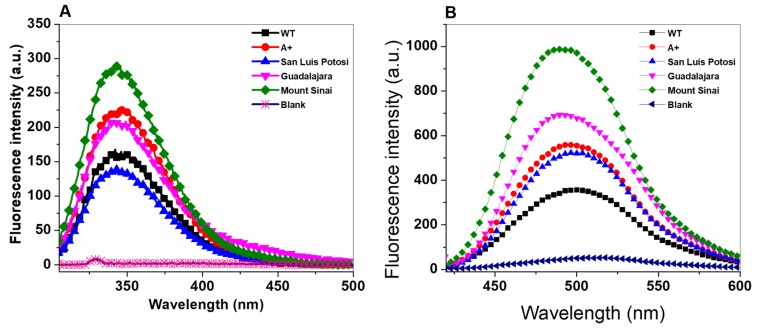
Fluorescence emission spectra of WT G6PD and variants. (**A**) Intrinsic fluorescence spectra and (**B**) 8-anilinonaphthalene-1-sulphonate (ANS) assays of WT G6PD and variants. Experimental conditions for all experiments are described in Materials and Methods.

To evaluate the effect on the single mutants G6PD A+ (N126D), San Luis Potosi (N126Y), and Guadalajara (R387C) and double mutant G6PD Mount Sinai (N126D/R387C) of structural alterations in the three-dimensional structure of the proteins and determine whether there was a correlation with the loss of catalytic activity, we performed intrinsic and extrinsic fluorescence assays. Intrinsic fluorescence assay was performed by monitoring the changes in intensity of the seventh tryptophan residues contained in the HuG6PD by monomer. We found that intrinsic fluorescence for the native WT G6PD enzyme showed a peak at 344 nm with a maximum intensity of 157 arbitrary units (a.u.) (Figure 7A), while the three clinical variants showed increased fluorescence intensity with respect to WT G6PD. The fluorescence intensity for Class III G6PD A+ was increased 1.4-fold (223 a.u.) compared to the WT G6PD enzyme, and for Class II G6PD San Luis Potosi (137 a.u.), G6PD Guadalajara (209 a.u.), and the double mutant G6PD Mount Sinai (286 a.u.) it increased 1.2-, 1.3-, and 1.8-fold, respectively, with respect to WT G6PD. These results are in concordance with fluorescence intensity obtained previously for Class I mutants G6PD Zacatecas and Durham, Class II G6PD Nefza, and double mutant G6PD A, respectively, where fluorescence intensity was increased two-fold [11,15]. As previously noted, increased fluorescence intensity could suggest modifications in the native 3D structure, which has a negative effect on the catalytic activity in the four clinical mutants.

To corroborate changes or alterations in the 3D structure of the WT G6PD protein provoke by G6PD A+, San Luis Potosi, Guadalajara, and G6PD Mount Sinai, we used 8-anilinonaphthalene-1-sulfonate (ANS) assays as a fluorescent molecular probe (extrinsic fluorescence). As seen in Figure 7B, all maximum extrinsic fluorescence intensities obtained for the four G6PD variants were higher than the WT G6PD enzyme, which showed a maximal fluorescence emission spectrum of 354, while Class III G6PD A+ showed an ANS spectrum with maximal fluorescence intensity of 558 a.u., and a 1.5-fold increase compared to the WT G6PD was observed. The emission spectrum for Class II G6PD San Luis Potosi and Class I G6PD Guadalajara and Mount Sinai showed maximal fluorescence of 523 a.u., 693 a.u., and 988 a.u., respectively, corresponding to an increase of 1.8-, 2-, and 2.8-fold fluorescence intensity, respectively (Figure 7B). The increased fluorescence level suggests conformational changes on the four mutants exposing hydrophobic sites to the solvent.

### 2.6. Evaluation of Stability of the Variants

#### 2.6.1. Thermal Stability Analysis of Recombinant G6PD Variants

**Figure 8:**
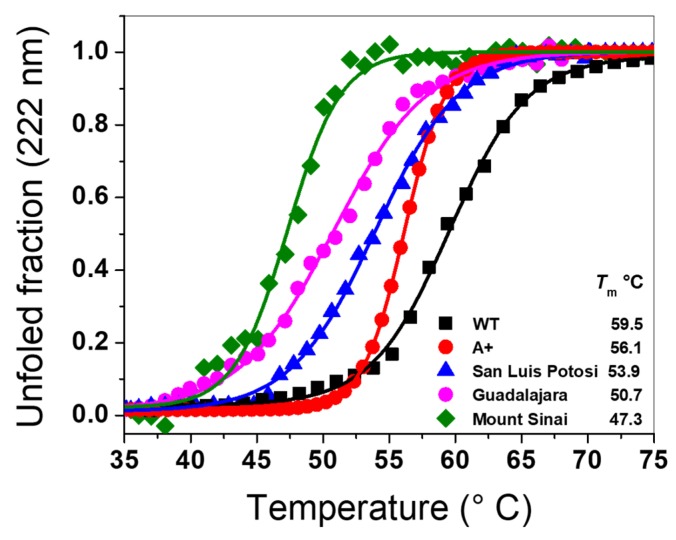
Analysis of structural stability of recombinant variants of G6PD. Thermal stability of the variants was followed by changes in DC signal at 222 nm through a temperature gradient (35–70 °C). Data were adjusted to Boltzman’s sigmoid equation in Origin 8.0 to obtain *T*_m_ value. The experiment was conducted in triplicate.

To analyze the effect of mutations on the structural stability of G6PD in the single mutants G6PD A+, San Luis Potosi, Guadalajara, and the double mutant G6PD Mount Sinai, we determined the changes in the structure of G6PD by DC at 222 nm, measuring the unfolded fraction of the α-helices through a temperature gradient (35–75 °C). The thermal denaturation of the G6PD proteins was followed by calculated *T*_m_ values, defined as the temperature at which half the α-helices remain in the native state and the other half are in an unfolded state. As shown in Figure 8, the *T*_m_ value calculated for the WT G6PD enzyme was 59.5 °C. We also calculated the *T*_m_ values, which are: 56 1 °C, 53.9 °C, 50.7 °C, and 47.3 °C for the single mutants G6PD A+, San Luis Potosi, and Guadalajara, and the double mutant G6PD Mount Sinai, respectively. Also, we observed a loss in *T*_m_ value of 3 °C, 6°, 10 °C, and 12 °C for G6PD A+, San Luis Potosi, Guadalajara, and G6PD Mount Sinai with respect to WT G6PD enzyme. These results indicate that the four G6PD mutants are more susceptible to denaturation by the temperature because these mutations made the three-dimensional (3D) structure are less stables with respect to WT G6PD. Once again, we observed that the stability of G6PD Mount Sinai (loss of 12 °C) was mainly due to the effect of G6PD Guadalajara (loss of 10 °C), and a slight additive effect of G6PD A+ was observed (loss of 3 °C). Furthermore, we determine the folding free energy which is an important biophysical characteristic of proteins that reflects the overall stability of the 3D structure of macromolecules [23]. The change of folding free energy (WT- mutant (MT)) determined for the Class III G6PD A+ variant (N126D) was −1.022166 kcal/mol; while that for the Class II G6PD San Luis Potosi variant (N126Y) a value of 0.613107 kcal/mol was determined. Finally, we determined a change of folding free energy of −9.000645 kcal/mol for the Class I G6PD Guadalajara variant (R387C). These values of change of folding free energy for all the mutants indicate that the change in the arginine residue by cysteine 387 (Arg→Cys) located in the binding site of structural NADP^+^ of the 3D structure suggest that the mutation destabilizes the protein respect to WT G6PD enzyme. In addition, these changes of folding free energy obtained are in concordance with the results previously showed, where higher alterations in secondary structure, a higher increase both in intrinsic and extrinsic fluorescence assays and a lower thermal stability for the single natural mutant R387C (G6PD Guadalajara) was observed respect to single natural variants G6PD A+ (N126D) and G6PD San Luis Potosi (N126Y). Finally, we observed that the effect of the mutations on stability of the G6PD protein was proportional to the loss of catalytic activity, secondary structure, and thermal stability where the single G6PD A+ variant showed catalytic activity around 50% with respect to WT G6PD enzyme; while G6PD San Luis Potosi, G6PD Guadalajara, and G6PD Mount Sinai showed a loss in catalytic constant of around 90%–95%.

#### 2.6.2. Thermal Inactivation Assays

**Figure 9:**
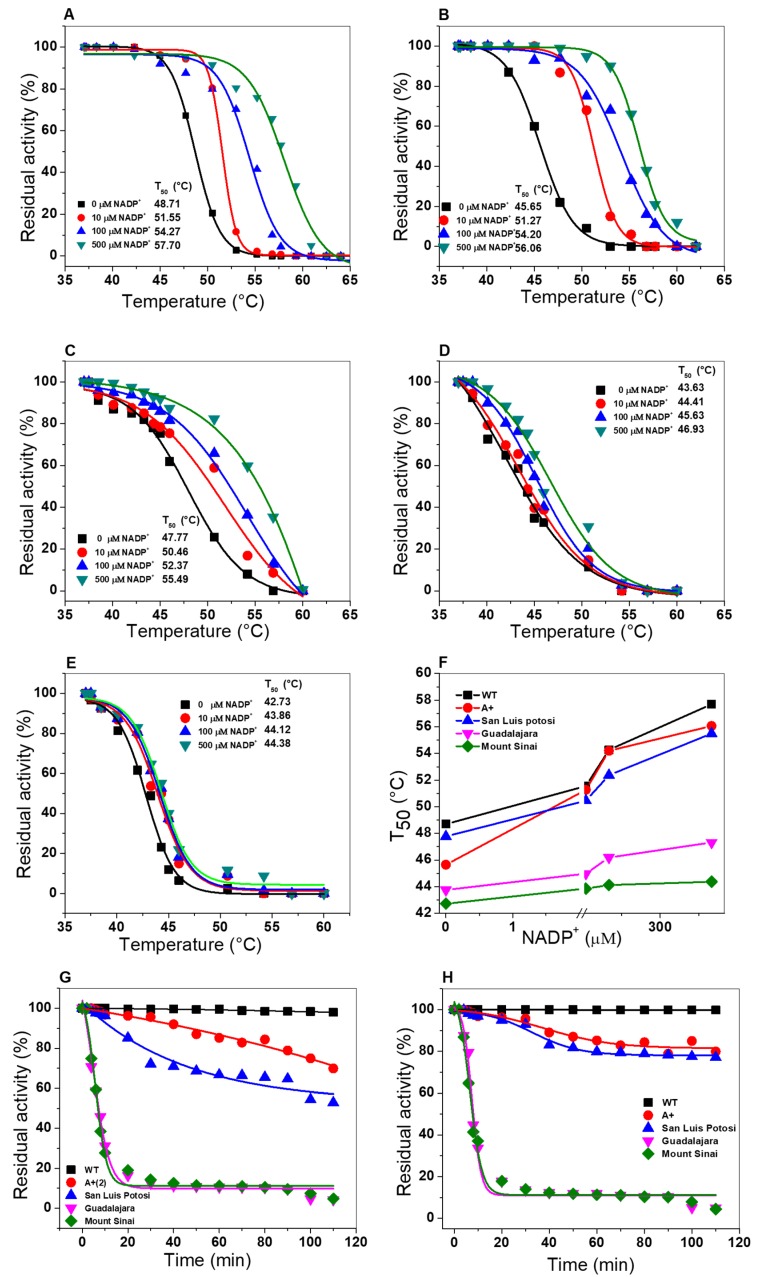
Thermal characterization of three single mutants and a double mutant of G6PD. (**A**) WT G6PD, (**B**) G6PD A+, (**C**) G6DP San Luis Potosi, (**D**) Guadalajara, and (**E**) G6PD Mount Sinai. The recombinant variants were incubated with different concentrations (0–500 µM) of NADP^+^ as a stability molecule for 2 h at 37 °C. Residual activity was plotted as percentage activity to calculate *T_50_* value of each mutant. (**F**) To determine changes in *T_50_* value of each variant, this was plotted against NADP^+^ concentrations. Thermal inactivation of G6PD variants through time (**G**) without NADP^+^ and (**H**) with 10 µM of NADP^+^. Assays were performed in triplicate.

To evaluate the effect caused by the mutations on the stability of the active site, thermal inactivation assays were performed for the single mutants G6PD A+, San Luis Potosi, and Guadalajara and the double mutant G6PD Mount Sinai. Thermal inactivation assays have been widely used to evaluate the effect caused by the point mutation on G6PD mutants in the absence or presence of different concentrations of NADP^+^ (0–500 µM) as a stabilizing agent, and *T_50_* (the temperature at which the enzyme loses 50% of its residual activity) was determined for each variant. Figure 9 shows the thermal inactivation assay for WT G6PD, G6PD A+, G6PD San Luis Potosi, G6PD Guadalajara, and G6PD Mount Sinai. In the absence of NADP^+^ molecules, the *T_50_* value determined for G6PD San Luis Potosi was 47.9 °C, while that for the WT G6PD enzyme was 48.7 °C (Figure 9A,B). Also, we determined a *T_50_* value of 45.3 °C for G6PD A+ (Figure 9C). These data suggest that there was no change in heat resistance for both enzymes with respect to WT G6PD enzyme. However, most thermolabile variants were G6PD Guadalajara (*T_50_* = 43.6 °C) and G6PD Mount Sinai (*T_50_* = 42.7 °C) (Figure 9D,E). On the other hand, when the concentration of NADP^+^ molecules increased (10, 100, and 500 µM), increased *T_50_* values were observed. The *T_50_* values at 500 µM NADP^+^ for WT G6PD, G6PD A+ (Class III), and G6PD San Luis Potosi (Class II) were 10 °C higher compared to the values obtained without NADP^+^ (Figure 9F). However, this increase is not reflected in the G6PD Guadalajara and G6PD Mount Sinai enzymes (both Class I variants), indicating that there is no protective effect by the NADP^+^ molecule because the mutations are located near the structural NADP^+^ binding in the native G6PD enzyme.

It is important to note that the stability of proteins by NADP^+^ toward the G6PD San Luis Potosi and A+ variants is in concordance with the protective function of NADP^+^ molecules that has also been observed in class I G6PD Yucatan [19], Zacatecas [11], and Class II G6PD mutants Viangchan [11], Vanua lava [11], Union [20], and even these mutations are not located near the active site or the union of structural NADP^+^. However, the Class I variants G6PD Guadalajara and G6PD Mount Sinai described in this study are consistent with reports of other Class I variants (G6PD Nashville [24], Durham [25], Wisconsin [24], Fukaya [26], and Campinan [26]), for which a protective effect was not observed when the concentrations of NADP^+^ was increased, because the mutations were located near the interface of the dimer and the structural NADP^+^ binding site of the native G6PD protein. In addition, these results are related with studies where has been proposed that an instability of G6PD could be a frequent effect of the deleterious mutation explaining the clinical manifestations of G6PD deficiency.

The loss of activity to different times was used for evaluate the possible changes caused by mutations in all the variants. These trials were carried out at a constant temperature (37 °C) and in the absence or presence of 10 µM of NADP^+^ as a stabilizing agent. As expected, the G6PD Guadalajara and Mount Sinai enzymes were the most thermolabile, showing loss of activity from more than 90% (Figure 9G). Likewise, the protein G6PD San Luis Potosi had a loss of activity near 60%; less affected was the G6PD A+ variant, with a loss of around 40%. When the variants were incubated with the physiological NADP^+^ concentration (10 µM), the protective effect was observed only on G6PD San Luis Potosi and G6PD A+. Despite that, the class I G6PD Guadalajara and G6PD Mount Sinai variants were again the most affected without changes in activity percentage compared with the WT G6PD (Figure 9H).

#### 2.6.3. Stability Analysis of G6PD Variants in the Presence of Guanidine Hydrochloride

**Figure 10:**
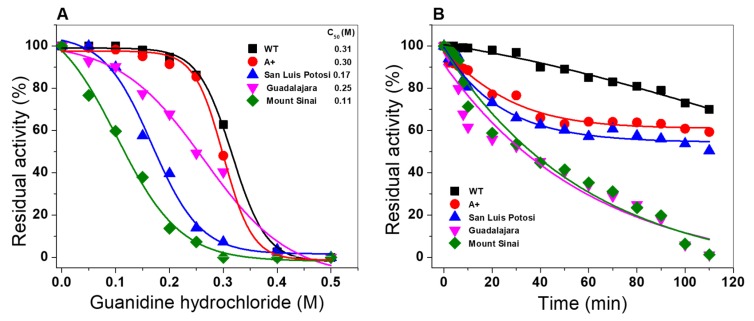
Stability analysis of recombinant human WT G6PD and four variants. (**A**) WT G6PD enzyme and G6PD A+, G6PD San Luis Potosi, G6PD Guadalajara, and G6PD Mount Sinai variants in the presence of guanidine hydrochloride (Gnd-HCL). Residual activity of enzyme expressed as percentage of activity for the same enzyme incubated at 25 °C in the absence of Gdn-HCl. (**B**) Inactivation of WT G6PD and mutants by 0.31M at 37 °C. At indicated times, aliquots were withdrawn from samples and assayed for residual activity. Both assays were performed in triplicate and standard errors were less than 4%.

The effect of the mutations on the stability of the WT G6PD enzyme was evaluated in the presence of chemical denaturant guanidine hydrochloride (Gdn-HCl). This assay has been used to determine the conformational stability of proteins because this alters the tertiary structure and therefore the catalytic activity is disturbed. As shown in Figure 10, a sigmoidal decay of the residual activity of the WT G6PD enzyme and the single natural variants G6PD A+, G6PD San Luis Potosi, and G6PD Guadalajara and double mutant Class I G6PD Mount Sinai decreased when the concentration of Gnd-HCl was increased. From the plot, we calculated the C_1/2_ values, which lets us calculate the values of C_1/2_ (concentration of Gnd-HCl at which the protein loses 50% of its residual activity at 2 h of incubation at 37 °C). The C_1/2_ values obtained were 0.31 M for the WT G6PD enzyme, and 0.30, 0.17, 0.25, and 0.11 M, respectively, for G6PD A+, G6PD San Luis Potosi, G6PD Guadalajara, and Mount Sinai (Figure 10A). As expected, the WT G6PD enzyme was most stable in the presence of Gnd-HCl. However, we observed that the single natural variant Class III G6PD A+ presented similar behavior to the WT G6PD enzyme because their C_1/2_ values are similar. On the other hand, it can be inferred the variant G6PD A+ (N126D) by itself does not show a loss of stability compared with the wild enzyme, but analyzing the mutation (A376T) originating from G6PD San Luis Potosi (N126Y), it is denaturalized 1.8 times faster than the WT G6PD enzyme, losing around of 90% of its residual activity when incubated at 0.3 M Gnd-HCl for 2 h; therefore, the mutation’s effect is to make it more susceptible to decreased residual activity due to denaturation. For G6PD Guadalajara and Mount Sinai, we observed a loss in residual activity of 1.2 and 2.8 times compared with the WT G6PD enzyme. These results again suggest that the double mutant G6PD Mount Sinai and single natural variant G6PD Guadalajara are probably more affected by the loss of catalytic activity and structural stability than the single mutants G6PD A+ and G6PD San Luis Potosi with respect to WT G6PD enzyme.

Moreover, based on the stability analysis of G6PD variants in presence of Gnd-HCl, we decided to evaluate the loss of activity in a time-course inactivation of WT G6PD and the four variants (0 to 120 min) incubated with Gdn-HCl_1/2_ (0.31 M) in the absence or presence of NADP^+^ molecules at a physiologic concentration (10 µM). The results show that for all G6PD mutants, a single exponential decrease time-course inactivation was obtained (Figure 9B). The enzymatic activity for the Class I G6PD Guadalajara and the double mutant G6PD Mount Sinai decreased rapidly, indicating that both were more susceptible to the Gnd-HCl, and they lost 100% of the initial activity after incubation with 0.31 M of the chaotropic agent for 90 min. However, the single mutants Class III G6PD A+ and Class II G6PD San Luis Potosi were the most resistant to Gnd-HCl because they showed a gradual loss in initial activity (60%) for 90 min.

#### 2.6.4. Susceptibility to Trypsin Digestion Assays

**Figure 11:**
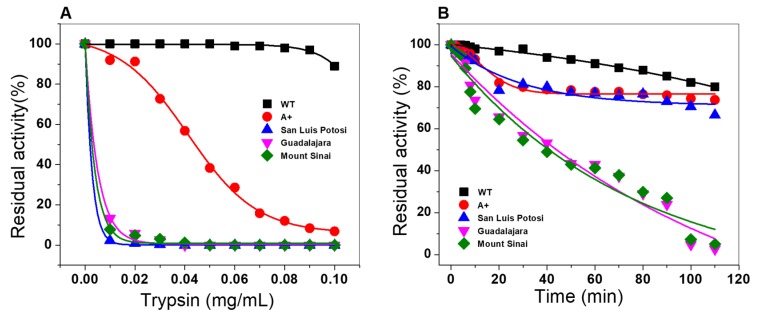
Evaluation of stability of G6PD proteins in trypsin digestion. Trypsin digestion of WT G6PD enzyme and single natural variants G6PD A+, G6PD San Luis Potosi, and G6PD Guadalajara and double mutant G6PD Mount Sinai in the (**A**) absence and (**B**) presence of NADP^+^ as stabilizing agent and different concentrations of trypsin.

To assess the effect of the mutations on protein stability, proteolytic susceptibility assays were performed with trypsin digestion protease in the absence or presence of physiological NADP^+^ concentration (10 µM). As seen in Figure 11A, when the three single natural variants and the double mutant were incubated with trypsin at different concentrations, all four variants became more susceptible to trypsin digestion, compared with the native enzyme (Figure 11A). The most susceptible variants were G6PD San Luis Potosi, G6PD Guadalajara and the double mutant G6PD Mount Sinai, where 100% of the initial activity was lost when the enzyme was incubated with 0.03 mg/mL of trypsin (6 units); the G6PD A+ variant showed more resistance to the trypsin digestion, losing 100% when it was incubated with 0.1 mg/mL of trypsin (19 units). It is interesting to note that at the same concentration of trypsin, WT G6PD showed residual activity of 100% as expected. These results indicate that the three single natural variants and the double mutant displayed high susceptibility to trypsin digestion compared to the native enzyme.

Nevertheless, with a physiologic concentration of NADP+ (10 µM) (Figure 11B), G6PD A+ and G6PD San Luis Potosi became more resistant to the digestion of the protease, indicating that the addition of NADP^+^ had a protective effect against trypsin digestion, whereas G6PD Guadalajara and G6PD Mount Sinai did not change based on the effect of resistance to digestion by NADP+, as these mutants were the most susceptible to protease degradation. These results agree with previously observed results in the thermal inactivation assay, where a protective effect was not observed when the concentration of NADP^+^ was increased, because the mutations were located near the interface of the dimer and the structural NADP^+^ binding site of the native G6PD protein (Figure 2). Furthermore, it is consistent with other Class I variants reported (G6PD Nashville [26], Durham [25], Wisconsin [24], Fukaya [26], and Campinan [26].

In addition, we observed again that the G6PD San Luis Potosi variant (Asn126Tyr) was more susceptible to trypsin digestion with respect to the G6PD A+ (Asn126Asp) variant, although both variants have the mutation in the same codon (126). According to a homology model of each mutant, we hypothesize that this difference in trypsin susceptibility occurs because the density of the ring of tyrosine residue could be interacting with structural neighbors with the chemical characteristics necessary to destabilize that vicinity due to repulsions between charges.

## 3. Materials and Methods

### 3.1. In Silico Mutagenesis and Modeling

Mutations in the crystal structure of human G6PD (PDB entries 2BHL and 2BH9) at positions 126 and 387 were generated in silico using the standard rotamer library of Coot [27] (Figure. The mutant models were subjected to energy minimization using YASARA software [28] (Supplementary Materials files: A+_00017465_phenix-coot-0.pdb, Global_00017462_phenix-coot-2.pdb, Guadalajara_00017463_phenix-coot-0.pdb, San_Luis_Potosi_00017464_phenix-coot-0.pdb). The graphical representations were prepared with CCP4mg version 2.10.11 [29].

### 3.2. Protein Stability Prediction in 3D

To determine the alterations on 3D structure by the changes in the amino acid sequence we used the method to predict the Single Amino Acid Folding free Energy Changes (SAAFEC) webserver (http://compbio.clemson.edu/SAAFEC/) and calculate the changes of the folding free energy caused by missense mutations [23]. We used the structure of G6PD dimer (Protein Data Bank entries 2BH9) and the mutations were performed in the server.

### 3.3. Construction of Recombinant G6PD Variants by Site-Directed Mutagenesis

The four clinical variants, three single mutants (G6PD A+, G6PD San Luis Potosi, and G6PD Guadalajara) and one double mutant (G6PD Mount Sinai), were generated by site-directed mutagenesis as previously described [19,21]. Polymerase chain reaction (PCR) was performed in a thermocycler (Mastercycler Eppendorf) using the plasmid pET-HisTEVP-*g6pd* containing the full human *g6pd* gene (NM_001042351 access) as a template. To generate the desired mutations, we used specific mutagenic forward and reverse primers (Table Supplementary 1) [30,31], and two flanking primers (forward and reverse) containing a restriction site *Nde*I and *Bpu*11021 in the extreme 5′ and 3′, respectively. The desired mutations were obtained by superposition of the derived products of the first and second PCR round, as described previously [25], flanking *Nde*l forward and reverse *Bpu*11021 primers. All PCR products were analyzed by electrophoresis in 1% agarose gel, stained with GelRed (Nucleic Acid Gel, Biotium, Fremon, CA, USA), and visualized in a MultiDoc-It Digital Imaging System (UVP, Upland, CA, USA). Amplicons with the desired size (1545 pb) were purified with the QIAquick Gel Extraction Kit (QIAGEN) (Valencia, CA, USA). The PCR products of each construction were purified and ligated to pJET 1.2 vector (CloneJET PCR Cloning Kit; Thermo Scientific, Hudson, NH, USA) and transformed in competent *E. coli* BW25113 cells. To confirm the insert fidelity and design of the mutations in the *G6PD* gene, the plasmid DNA was isolated and bidirectional DNA sequencing with verified internal forward and reverse sequencing primers was performed. The pJET 1.2 vector containing the verified sequence for each mutant was digested with restriction enzymes *NdeI* and *Bpu11021*, then sub-cloned into the pET3a plasmid (Novagen, Madison, WI, USA) and identified as follows: pETgR387C, pETgN126Y, pETgN126D, and pETgN126D/R387C. Then, the constructions were transformed into competent *E. coli BL21(DE3)Dzwf ::kanr* [19].

### 3.4. Expression and Purification of G6PD Recombinant Variants

Recombinant expression of the single mutant G6PD A+, G6PD San Luis Potosi and G6PD Guadalajara, and the double G6PD Mount Sinai were expressed as previously described [11,19,25]. To obtain the most G6PD soluble protein, we determined the optimal expression conditions of each G6PD variant in 25 mL of Luria Bertani (LB) culture medium (25 °C) varying the concentration of isopropyl-β-D-thiogalactopyranoside (IPTG) and induction time (2, −18 h.). The cells were concentrated, harvested and broken. The crude extract was clarified and the supernatants were used to calculate specific G6PD activity. Later, we scaled the cell culture of G6PD variants to 2 L of LB medium with 100 µg/mL of Amp^r^ and Kan^r^ with the optimal condition expression of each G6PD variant (IPTG concentration and expression time). After the cell sonication, the crude extract was obtained removing the cell debris, and the supernatant was used to purify the enzymes.

Purification of G6PD enzymes was performed as previously described by Gomez-Manzo et al. [11,19,25]. The purity and concentration of G6PD proteins were determined by 12% SDS-PAGE gels and Lowry assay [32] (bovine serum albumin (BSA) was used as the standard). The G6PD proteins were used immediately after being purified.

### 3.5. Determination of Steady-State Kinetic Parameters

The kinetic parameters of the recombinant enzymes G6PD A+ (Asn126Asp), G6PD San Luis Potosi (Asn126Tyr), G6PD Guadalajara (Arg387Cys), and G6PD Mount Sinai (Asn126Tyr/Arg387Cys) were determined spectrophotometrically at 25 °C when monitoring the reduction of the NADP+ substrate into an absorbance of 340 nm [11,15,19,25,33]. Standard activity assay was performed in a final volume of 1 mL of the reaction in buffer T (Tris-HCl 0.1 mM, pH 8.0, and MgCl_2_ 3 mM). The initial velocity data for the G6P substrate was obtained by varying the substrate in a range from 2.5 to 200 µM while the second substrate (NADP^+^) was fixed at saturating concentration (1 mM); meanwhile the initial velocity data for NADP^+^ was obtained varying this substrate in a range from 2.5 to 200 µM while the second substrate (G6P) was fixed at saturating concentration (1 mM). The reaction was initiated with the addition of 200 ng/mL of each G6PD variant. The steady-state kinetic parameters, K*_m_*, *k*_cat_, and V*_max_* were calculated by fitting the data to the Michaelis–Menten equation (Vi = Vmax [S]/Km + [S]) by nonlinear regression calculation. *k*_cat_ was derived from Vmax considering the molecular mass of the monomer of 59.25 kDa. One international unit (UI) of G6PD enzymatic activity is the quantity of enzyme required to produce one µmol of NADPH for one minute. A standard activity assay was performed with a final volume of 1 mL and the standard reaction mixture (100 mM Tris-HCl buffer at pH 8.0, 3 mM MgCl_2_, 1 mM of G6P, and 1 mM NADP^+^).

### 3.6. Circular Dichroism (DC) Analysis

To evaluate the effects of the single and double mutants in the G6PD protein, we determined the secondary structure by circular dichroism (CD) analysis employing a spectropolarimeter (Jasco J-8190^®^, Easton, MD, USA) equipped with a Peltier thermostat cell holder. The WT G6PD and variants were adjusted at a final protein concentration of 0.2 mg/mL in 50 mM phosphate buffer at pH 7.35. Far UV-CD spectra (120–250 nm) of each G6PD protein were collected with 1 nm intervals on a path-length quartz cuvette of 1 nm. The far UV-CD spectra of the blank was subtracted from those containing the respective protein [11,15,25,33]. The experiments were performed in triplicate at 25 °C.

### 3.7. Structural Analysis by Intrinsic and 8-Anilinonaphthalene-1-Sulfonate (ANS) Binding Assays

To determine the effects of the four mutations on the tertiary structure of the proteins, intrinsic fluorescence assays and 8-anilinonaphthalene-1-sulfonate (ANS) binding assays were performed to monitor the conformations on the tertiary structure. Both assays were performed on a Perkin-Elmer LS-55 fluorescence spectrophotometer (Perkin Elmer, Wellesley, MA, USA) equipped with a quartz cell and a step length of 1 cm [11,15,25,33]. The intrinsic fluorescence assay of WT G6PD and the four variants was performed using a final protein concentration of 0.1 mg/mL variant in 50 mM phosphate buffer at pH 7.35 at 25 °C. The excitation wavelength used for the samples was 295 nm and fluorescence emission was obtained from 300–500 nm with 4.5 and 3.7 nm slits for excitation and emission, respectively. Background fluorescence from the buffer (blank) was subtracted from those containing the respective protein [11].

With respect to ANS binding assays, the proteins were adjusted to 0.4 mg/mL in 50 mM phosphate buffer at pH 7.35. The excitation wavelength used was 395 nm and emission spectra were recorded from 400–600 nm with 5 nm slits for excitation and emission. Background fluorescence from the buffer plus ANS was subtracted from those with respective proteins.

### 3.8. Evaluation of Stability of the Variants

#### 3.8.1. Thermal Stability Analysis of Recombinant G6PD Variants

The effects of the mutations were determined by thermal stability. The unfolding of the WT G6PD and variants were determined following the changes in molar ellipticity at 222 nm as the temperature was varied from 20–75 °C at a rate of 1 °C/2.5 min. Before the assay, the structural NADP^+^ of the proteins was removed as previously reported [15,19,33], then the WT G6PD and four variants were adjusted at 0.2 mg/mL. Melting temperature (*T_m_*) was defined as the fraction at which 50% of the protein was folded/unfolded and was calculated as previously reported [11,15,28].

#### 3.8.2. Thermal Inactivation Assays

Thermal inactivation assays are used as an indicator of the stability of the G6PD enzyme based on the residual activity when they are exposed to different temperatures and NADP+ concentrations as a stabilizing agent. To perform this assay, we removed the structural NADP^+^ molecule as previously reported [15,19,33]. The WT G6PD and the four variants were adjusted at 0.2 mg/mL and incubated with three concentrations of NADP^+^ (10, 100, and 500 µM) in a thermocycler (Biorad) for 20 min to different temperatures (37 to 60 °C), after the samples were cooled to 4 °C in 5 min increments. Later, residual G6PD activity was measured with a standard activity assay and expressed as percentage of activity. Residual activity of the enzyme incubated at 37 °C was fixed at 100%. All thermal inactivation assays were performed in triplicate.

#### 3.8.3. Stability Analysis of G6PD Variants in the Presence of Guanidine Hydrochloride

This section may be divided by subheadings. It should provide a concise and precise description of the experimental results, their interpretation as well as the experimental conclusions that can be drawn.

#### 3.8.4. Thermal Stability Analysis of Recombinant G6PD Variants

The stability of WT G6PD and the four variants were evaluated in the presence of guanidine hydrochloride (Gdn-HCl) to evaluate the effect of the mutations on the stability of the proteins. All proteins were adjusted to 0.2 mg/mL in 50 mM phosphate buffer at pH 7.35 and incubated with different concentrations of chaotropic agent Gdn-HCl (0.05, 0.10, 0.15, 0.20, 0.25, 0.30, 0.40, 0.5, and 1 M) at 37 °C for 2 h, then the residual activity was measured spectrophotometrically at 340 nm with a standard activity assay at 25 °C. The residual activity of the enzyme incubated at 37 °C without the denaturing Gdn-HCl chaotropic agent was fixed at 100%. The assay was performed in triplicate.

#### 3.8.5. Susceptibility to Trypsin Digestion Assay

Another approach to evaluate stability is the trypsin digestion assay. We performed this assay in the absence and presence of NADP^+^ molecules at the physiological concentration (10 µM). The G6PD proteins were adjusted to a final concentration of 0.2 mg/mL and incubated with trypsin (0.5 mg/mL) at 37 °C for 2 h as previously reported by Cortes-Morales et al. [33]. Later, residual enzymatic activity was measured with a standard activity assay and expressed as percentage of activity. The residual activity of the enzyme incubated without trypsin was fixed at 100%. The assay was performed in triplicate.

## 4. Conclusions

Functional and structural analysis of three single natural variants G6PD A+ (N126D) (Class III), G6PD San Luis Potosi (N126Y) (Class II), and G6PD Guadalajara (R387C) (Class I) and double mutant Mount Sinai (N126D/R387C) (Class I) showed alterations in the catalytic activity, secondary structure, and global stability and changes in the three-dimensional (3D) structure of the protein with respect to WT G6PD enzyme. Furthermore, based on the functional and structural results, we suggest that the single variant Class II G6PD San Luis Potosi could be reclassified as a variant class I considering the clinical and biochemical characteristics, as proposed by Luzzato and coworkers [1] because this variant showed a loss of catalytic efficiency of around 95% and changes in stability as in Class I (Zacatecas, Durham, Nashville) variants as previously reported [11,24,25]. This reclassification and biochemical characterization of the G6PD recombinant variants could provide information about the environment of mutation in the structure and subsequently develop molecules that stabilize or reset the lost interactions that cause G6PD variants and possibly prevent clinical manifestations.

## Figures and Tables

**Table 1:** Purification summary of recombinant human WT G6PD and four variant enzymes.

G6PD	Amino Acid Substitution and Class	Total Protein (mg)	Specific Activity (IU.mg^−1^)	Total Activity (IU)	Yield (%)
WT	-	2.1	230	483	61
A+	N126D (III)	1.8	114	205	43
San Luis Potosi	N126Y (II)	0.4	15	6	2
Guadalajara	R387C (I)	1.8	15	27	3
Mount Sinai	N126D/R387C (I)	1.8	16	29	2

Roman numerals indicate variant classes.

**Table 2:** Summary of kinetic parameters of WT G6PD and four G6PD mutants.

G6PD	*Class*	*Amino Acid Substitution*	*k*_cat_ (s^−1^)	*K*_mG6P_ (µM)	K_mNADP_^+^ (µM)	*k*_cat_/*K*_mG6P_ (s^−1^ M^−1^)	*k*_cat_/*K*_mNAD*P*_*^+^* (s^−1^ M^−1^)
WT	-		230	38.4	6.1	5.9	37.3
A+	III	Asn126Asp	114	56.4	12.9	2.0	8.7
San Luis Potosi	II	Asn126Tyr	10.4	43.7	11.9	1.35	0.28
Guadalajara	I	Arg387Cys	25.9	98.8	41.4	0.15	0.37
Mount Sinai	I	Asn126Asp/ Arg387Cys	13.9	27.1	41.6	0.59	0.38

## Supplementary Materials

Supplementary materials can be found at https://www.mdpi.com/1422-0067/21/8/2732/s1. Table S1: List of primers used in this study.
