# Supplementary material for: Effects of Single and Double Mutants in Human Glucose-6-Phosphate Dehydrogenase Variants Present in the Mexican Population: Biochemical and Structural Analysis
Source: Int J Mol Sci. 2020 Apr 15;21(8):2732. doi: 10.3390/ijms21082732 (PMC7215812; doi:10.3390/ijms21082732)
Supplement: Supplementary file 1 [file ijms-21-02732-s001.zip › Supplementary Materials/Supplementary File.docx]

**Supplementary file**

Table Supplementary 1. . List of primers used in this study.

| **Strain *E. coli*** | **Relevant Characteristic(s) or Sequence** | **Source and/or Reference** |
| --- | --- | --- |
| BW25113 | F^−^, DE(araD-araB)567, lacZ4787(del)::rrnB-3, LAM^−^, rph-1, DE(rhaD-rhaB)568, hsdR514 | [25] |
| BL21(DE3)Δ*zwf*::*kan^r^* | F^−^ ompT gal dcm lon hsdS_B_(r_B_^−^ m_B_^−^) λ(DE3 [lacI lacUV5-T7 gene 1 ind1 sam7 nin5]) Δ*zwf-777*::*kan*. | [15] |
| **Plasmids** |  |  |
| pET-HisTEVP-*g6pd* | pETg6pd carrying the human *g6pd* gene, *Amp^R^* | [15] |
| pJETg6pd A+ | pJET 1.2 plasmid carrying the human *g6pd* gene with a Asn126Asp mutation in the G6PD protein, *Amp^R^* | This study |
| pJETg6pd San Luis Potosí | pJET 1.2 plasmid carrying the human *g6pd* gene with a Asn126Tyr mutation in the G6PD protein, *Amp^R^* | This study |
| pJETg6pd Guadalajara | pJET 1.2 plasmid carrying the human *g6pd* gene with a mutation of Arg387Cys in the G6PD protein, *Amp^R^* | This study |
| pJETg6pd Mount Sinai | pJET 1.2 plasmid carrying the human *g6pd* gene with a double mutation of Asn126Asp + Arg387Cys in the G6PD protein, *Amp^R^* | This study |
|  |  |  |
| pETg6pd A+ | pET-3a carrying the human *g6pd* gene with a Asn126Asp mutation in the G6PD protein, *Amp^R^* | This study |
| pETg6pd San Luis Potosi | pET-3a carrying the human *g6pd* gene with an Asn126Tyr mutation in the G6PD protein, *Amp^R^* | This study |
| pETg6pd Guadalajara | pET-3a carrying the human *g6pd* gene with a mutation of Arg387Cys in the G6PD protein, *Amp^R^* | This study |
| pETg6pd Mount Sinai | pET-3a carrying the human *g6pd* gene with a double mutation of Asn126Asp + Arg387Cys in the G6PD protein, *Amp^R^* | This study |
| **Mutagenesis** | **Primer Sequence** |  |
| A+ fw | 5- AGCCACATG**G**ATGCCCTCCAC -3′ | This study |
| A+ rev | 5′- GTGGAGGGC**A**TCCATGTGGCT -3′ | This study |
| San Luis Potosí fw | 5´ AGCCACATG**T**ATGCCCTCC 3´ | This study |
| San Luis Potosí rev | 5´ GGAGGGCAT**A**CATGTGGCT 3´ | This study |
| Guadalajara fw | 5´ CAGTGCAAG**T**GCAACGAGC 3´ | This study |
| Guadalajara rev | 5´ GCTCGTTGC**A**CTTGCACTG 3´ | This study |
| **Oligonucleotides for Sequencing** | **Primer Sequence** |  |
| Flanking *NdeI* fw | 5- CGACAGC**CATATG**GCAGAG-3′ | This study |
| Flanking *Bpu* rev | 5′- TGC**GCTGAG**CTCAGAGCTT -3′ | This study |
| Internal G6PD fw | 5'-GGCCAACTGCCTCTTCTAC-3' | This study |
| Internal G6PD rev | 5'-GAGAAGGTCAAGATGTTGAAATG-3' | This study |

Locations of mutagenic oligonucleotides are in bold.
